# Insulin-like growth factor-1 improves *in vitro* meiotic resumption of dromedary camel (Camelus Dromedarius) oocytes

**DOI:** 10.1590/1984-3143-AR2022-0105

**Published:** 2023-06-19

**Authors:** Abdulrahman Khalid Alhaider

**Affiliations:** 1 Department of Clinical Sciences, College of Veterinary Medicine, King Faisal University, Alahsa, Saudi Arabia

**Keywords:** camel, cathepsin B, insulin-like growth factor I, IVM, oocytes

## Abstract

Despite relatively high maturation rate of *in vitro* matured oocytes in the dromedary camel, however, blastocyst production is very low after *in vitro* fertilization (IVF). Herein, the influences of oocyte collection method (follicular aspiration *vs* slicing; *Experiment I*), the addition of Insulin-like growth factor I (IGF-I) to the maturation medium (*Experiment II*) on *in vitro* maturation (IVM) of oocyte were investigated. Although the nuclear maturation did not differ regardless of collecting method, follicular aspiration led to lower degeneration rates than those in controls (P < 0.05). The percentages of oocytes at MII were greater in the presence of IGF-1 than in its absence (71.9% vs 48.4%, respectively, P<0.05). Additionally, the percentages of degenerated oocytes were higher in the control group compared to oocytes cultured in the presence of IGF-I (23.6% *vs* 10.4%, respectively, P<0.05). IGF-I treatment improved the quality of MII matured oocytes as evidenced by the decrease of cathepsin B (CTSB) activity, a marker of poor quality oocytes, when compared to control ones (P < 0.05). In conclusion, follicular aspiration decreased the degeneration rate; however, it had no effect on completion of maturation. IGF-I enhanced the IVM of oocyte and decreased degeneration rate.

## Introduction

There hasn't been as much research done on dromedary camel reproductive issues as there has been on cattle ones (El-Deeb et al., 2022). Assisted reproductive technologies (ARTs) including *in vitro* maturation (IVM), the *in vitro* fertilization (IVF) of oocytes, and *in vitro* culture (IVC) for the production of transferable embryos have been regarded as a propitious strategy to strive against infertility issues (Szamatowicz, 2016; Kushnir et al., 2017).

Several supplements to the culture medium have been used to improve the outcome of IVM in the dromedary such as gonadotrophins (Abdoon et al., 2007; Khatir et al., 2007), serum (Abdoon, 2001; Khatir and Anouassi, 2006), proteins Wani and Wernery, 2010), leptin (Gabr et al., 2014), and caffeine (Fathi et al., 2014).

Growth factors have an autocrine and paracrine regulatory role in ovarian function (Arat et al., 2016). Growth factors include among others, growth hormone (GH), Insulin-like Growth Factor I (IGF-I), Transforming Growth Factor-α (TGF-α), Fibroblast Growth Factor (FGF), and Epidermal Growth factor (EGF). IGF-I has been demonstrated to promote cellular mitosis and differentiation in a variety of systems (Giudice, 1992). In the ovary, IGFs stimulate granulosa cell proliferation, aromatase activity, and progesterone biosynthesis (Adashi et al., 1985; Kamada et al., 1992). IGF-I stimulated the nuclear maturation of oocytes in humans (Gómez et al., 1993), bovines (Lorenzo et al., 1994), rabbits (Lorenzo et al., 1996), and canines (Sato et al., 2018). The addition of IGF-1 to the IVM medium also increased the maturation rate, and improved IVF results in Additionally, IGF-I enhanced blastocyst rate in mice (Demeestere et al., 2004) and swine (Oberlender et al., 2013). Moreover, IGF-I has anti-apoptotic activity in in vitro matured oocytes in rabbits (Herrler et al., 1998, and bovine (Wasielak and Bogacki, 2007).

Cathepsin B (CTSB) is a lysosomal cysteine protease that is found in several types of cells such as bovine oocytes (Balboula et al., 2010). Inhibition of CTSB during IVM significantly improved the developmental competence of bovine COCs and the quality of their embryos (Balboula et al., 2010). Moreover, the activity of CTSB was found to be correlated inversely with the developmental competence of bovine oocytes after in vitro grown oocytes (IVG) obtained from the early antral follicle (Abdel-Ghani et al., 2019), that CTSB activity can be helpful to use as a marker for inferior quality oocytes (Abdel-Ghani et al., 2019). Although the role of CTSB activity has been elucidated in bovine oocytes, no available data exists regarding its activity in camel oocytes after IVM.

Therefore, the present study was designed to evaluate the influence of the collection method on dromedary camel oocytes' nuclear maturation, and the effects of IGF-1 supplementation during IVM on dromedary camel oocytes maturation and the quality of the oocytes after IVM (CTSB activity).

## Methods

### Chemicals

All the chemicals used in the present study were purchased from Sigma-Aldrich (St. Louis, MO, USA), unless otherwise stated.

### Animals

Camel ovaries were obtained from a local abattoir (Alahasa, Saudi Arabia) during the breeding season (December to February). Both ovaries from each camel were transported to the laboratory within 1 to 2 h in a thermos flask at 25 °C containing physiological sterile saline supplemented with 100 IU/mL penicillin and 100 μg/ml streptomycin sulphate. After three washes in physiological sterile saline, the ovaries were placed in phosphate buffered saline (FBS) supplemented with penicillin/streptomycin until processing.

The study was approved by the Veterinary Teaching Hospital's Animal Care Committee, King Faisal University, Saudi Arabia (No. DSR-10044).

### Collection of cumulus oocyte complexes (COCs)

#### Aspiration method

**Figure 1:**
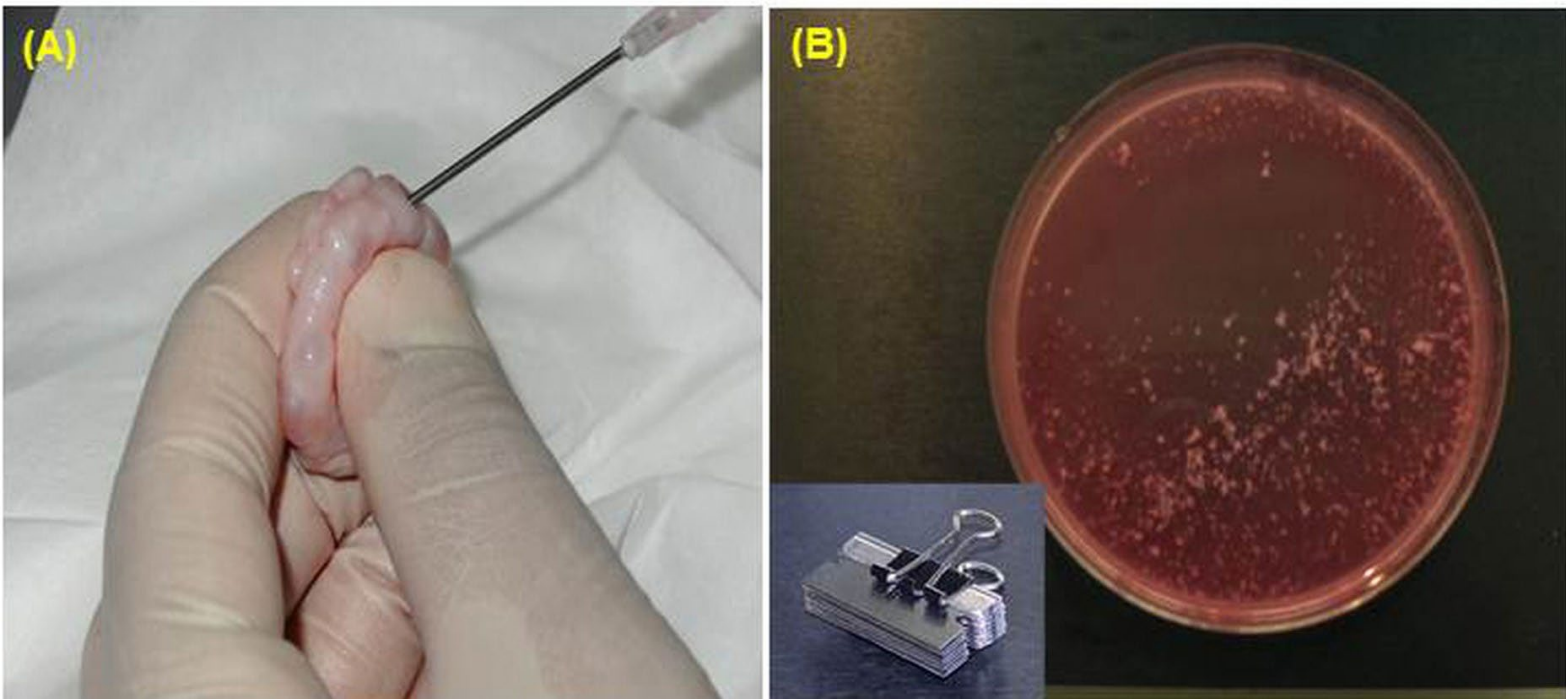
Collection of oocytes by follicular aspiration (A) or slicing with multiple blades (B).

Follicles (3-10 mm in diameter) were aspirated using an 18-gauge needle attached to a 10-ml syringe containing working medium (HEPES buffered-TCM 199) supplemented with 10% (vol/vol) fetal calf serum (FCS) (Figure 1A). Aspirated follicular fluid containing oocytes was pooled in 50-mL conical tubes containing working medium, and maintained at 39 ˚C for 10 minutes, allowing the COCs to settle to the bottom of the tubes.

#### Slicing method

The COCs were released by repeatedly slicing the ovarian cortex to <1 mm with multiple razor blades in 90 mm petri dishes containing 10 to 15 ml working medium (Figure 1B).

#### COCs selection

COCs were searched for under a stereomicroscope and washed three times in a four well dish (Nunc A/S) containing working medium. Those with more than three layers of cumulus cells and a uniform homogeneous cytoplasm were selected for further processing.

#### IVM of COCs

The collected oocytes were submitted to IVM as described previously (Khatir et al., 2005). Briefly, COCs were incubated in IVM medium (approximately 10 COCs/100 μL) and were then covered with paraffin oil for 30 h at 39 °C in a humidified atmosphere with 5% CO2. Maturation medium consisted of HEPES-buffered TCM199 supplemented with 50 μg/mL sodium pyruvate, 10 μg/mL FSH (from the porcine pituitary), 10% FCS, and μg /mL gentamicin sulfate.

#### Evaluation of oocyte nuclear maturation

**Figure 2:**
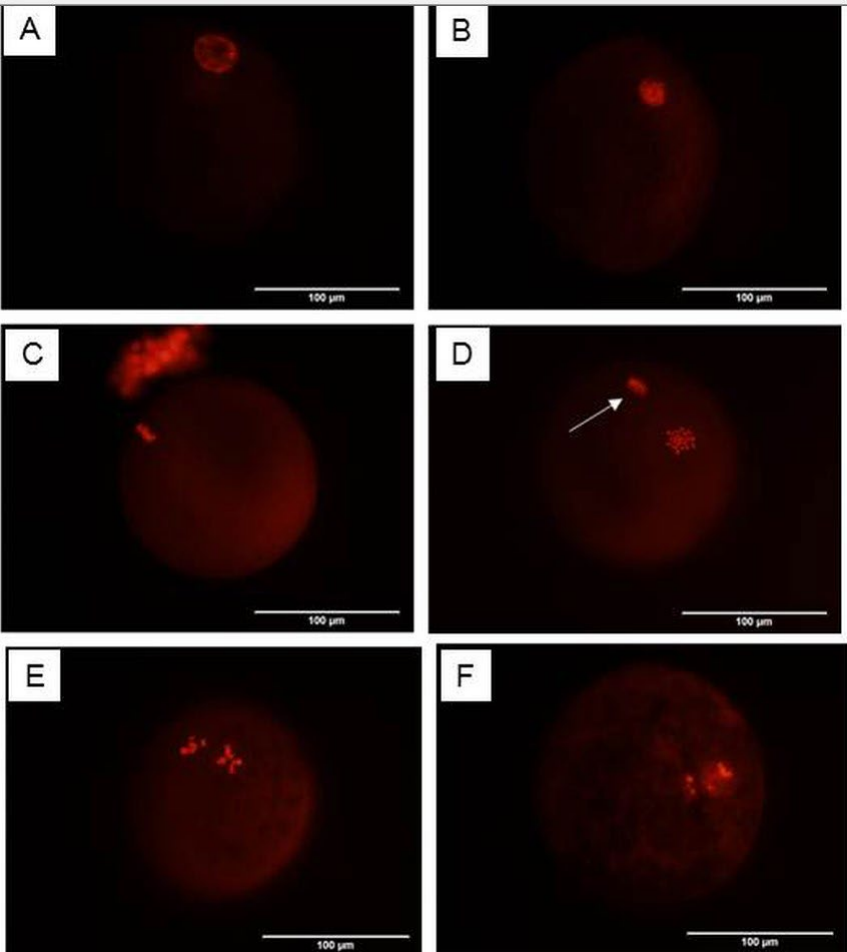
Fluorescence photomicrographs show chromatin configuration in camel oocytes stained with propidium iodide (PI stain). (A) Immature oocyte having germinal vesicle; (B) Germinal vesicle break down; (C) Metaphase I; (D) Mature oocyte having metaphase II plate (MII) (white arrow) and a polar body (arrowhead); (E, F) Degenerated oocyte. (×400).

Following IVM, oocytes were denuded from cumulus cells by vortexing three min in 1% warm sodium citrate solution (1 ml) (Alhaider and Watson, 2009). Oocytes were fixed in 2% paraformaldehyde / 0.25% Triton X- 100 in PBS for 30 min at 39 °C followed by 30 min in 4% paraformaldehyde/ 0.25% Triton X-100/ PBS at 4 °C. Then, they were washed three times in PBS supplemented with 1% BSA. The cumulus-free oocytes were stained with 10 µg/mL propidium iodide (PI) in PBS and incubated for 15 min in darkness. Afterwards they were washed again three times in PBS and placed on glass slides. The oocytes were subsequently overlaid with a coverslip. The chromatin state were evaluated using a fluorescence microscope with UV light (Olympus, Japan) to determine the meiotic stage following Wani and Nowshari (2005); as follows: (a) immature or germinal vesicle (GV, Figure 2A), when the nucleolus was surrounded by condensed chromatin; (b) resumption of meiosis or germinal vesicle break down (GVBD, Figure 2B), when the chromatin was dispersed; (c) metaphase I (M-I, Figure 2C), when chromosomes were highly compact in a metaphasic plate and migrating to the poles; (d) mature (metaphase II; M-II, Figure 2D), when chromosomes were in second metaphase with the extrusion of the first polar body; (e) degenerated (deg, Figure 2E-2F) when oocytes with loss of membrane integrity or showing dispersed chromosomes.

#### Evaluation of CTSB activity after IVM oocytes

The detection of CTSB activity in COCs derived from OCGCs was performed using the Magic red CTSB detection kit (P 6133; Immunochemistry Technologies LLC, Bloomington, MN, USA) according to the manufacturer’s instructions and as previously described (Balboula et al., 2010). Briefly, COCs after IVM were incubated in 500 μL DPBS with 2 μL of the reaction mix in a 4-well dish (176740 Nunc, Thermo Fisher Scientific, Roskilde, Denmark) in a humidified atmosphere of 5% CO_2_ at 38.5 °C for 20 min. Hoechst 33342 was added at a concentration of 25 µg/ml to detect nuclei, and incubated under the same culture conditions for a further 10 min. After rinsing in DPBS containing 3 mg/ml PVP, stained fresh oocytes were mounted onto a glass slide with a coverslip, and examined under the fluorescence microscope (LEICA). An excitation filter of 365 nm was used to detect nuclei, while an excitation filter of 550 nm was applied to observe CTSB activity. CTSB activity images of oocytes were captured and analyzed by ImageJ software (NIH).

### Experimental design

#### Experiment I

A total of 509 oocytes (117 ovaries) were used to evaluate the effect of oocytes harvesting method either using follicular aspiration (n= 237; 8 replicates) or slicing method (n= 272; 8 replicates) on nuclear maturation of dromedary oocytes.

#### Experiment II

A total of 189 oocytes (48 ovaries) were used in order to evaluate the presence of IGF-I (n = 96; 8 replicates) or absence (n = 93; 8 replicates) supplementation on nuclear maturation of dromedary oocytes. In the IGF-I treated group, 100 nM^-1^ of recombinant human IGF-I was added to maturation medium. Maturation medium without IGF-I was used as a control.

#### Experiment III

To evaluate the effect of IGF-I on the quality of oocytes after IVM, the CTSB activity (3 replicates) was investigated using a total of 60 oocytes derived.

### Statistical analysis

All statistical analyses were performed using the procedures of SPSS release 12.0.1 software (SPSS Inc., Chicago, IL, USA). A Chi-square test (two-tailed) with Yates correction for continuity when appropriate was used to evaluate individual specific factorial arrangements. Differences of P < 0.05 were regarded as significant.

## Results

### Experiment I: effect of oocytes harvesting method (follicular aspiration vs slicing) on nuclear maturation of camel oocytes

The results are shown in Table 1. In aspirated oocytes, the proportions of oocytes at GV were higher than those collected by slicing (P < 0.05). However, the proportion of degenerated oocytes was higher (P < 0.05) when oocytes were collected by the slicing method.

**Table 1:** Effects of collecting method on the nuclear status of oocytes after IVM for 30 h.

**Collecting method**	**No. of ovaries**	**No. of oocytes (replicates)**	**No. of oocytes in each meiotic stage (%)**					
			**GV GVBD MI MII Deg. GVBD-MII**					
Aspirated follicle	61	237 (8)	29 (12.2)^a^	9 (3.8)	28 (11.8)	118 (49.8)	53 (22.4)^a^	155 (64.4)
Slicing	56	272 (8)	10 (3.7)^b^	7 (2.3)	46 (16.9)	128 (47.1)	102 (37.5)^b^	161 (59.2)

GV: germinal vesicle; GVBD: germinal vesicle breakdown; MI: metaphase I; MII: metaphase II. a-b: Within a column, values without a common superscript significantly differed (P < 0.05).

### Experiment II: effect of IGF-I supplementation on nuclear maturation of camel oocytes

The results for the IVM of oocytes in the presence and absence of IGF-I are shown in Table2. The number of oocytes at the M II stage and overall meiotic resumption were higher in the IGF- I group than in the control group (P < 0.05). No differences were observed in GVBD and MI rates between the groups (P > 0.05). However, the rate of oocyte degeneration was lower in the IGF-I-treated group (P 0.05).

**Table 2:** Effects of IGF-I supplementation on the nuclear status of oocytes after IVM for 30 h.

**Treatment**	**No. of oocytes (replicates)**	**No. of oocytes in each meiotic stage (%)**					
		**GV GVBD MI MII Deg. GVBD-MII**					
Control	93 (8)	10 (10.7)^a^	4 (4.3)	12 (12.9)	45 (48.4)^a^	22 (23.6)^a^	61 (65.6)^a^
IGF-I	96 (8)	4 (4.2)^b^	5 (5.2)	8 (8.3)	69 (71.9)^b^	10 (10.4)^b^	85 (88.5)^b^

GV: germinal vesicle; GVBD: germinal vesicle breakdown; MI: metaphase I; MII: metaphase II. a-b: Within a column, values without a common superscript significantly differed (P < 0.05).

### Experiment III: effect of IGF-I treatment on CTSB activity

**Figure 3:**
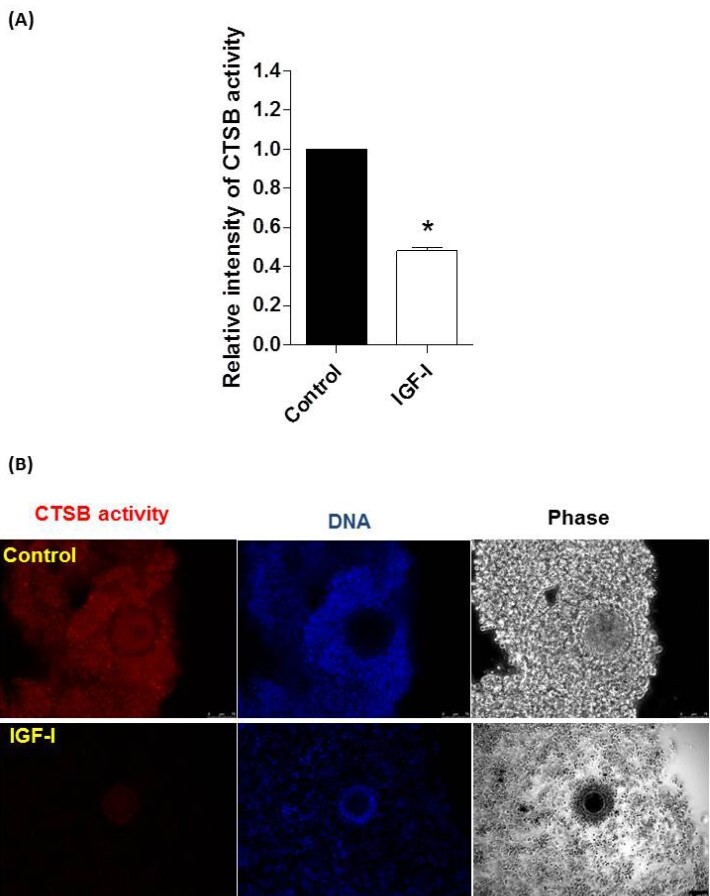
Effect of absence and presence of Insulin-like Growth Factor I (IGF-I) to IVM medium on cathepsin B (CTSB) activity in cumulus-oocyte complexes (COCs). Quantification of the relative fluorescence intensity of CTSB activity (A). CTSB were detected as red fluorescence dots (B). An excitation filter of 365 nm was used to detect nuclei, while an excitation filter of 550 nm was applied to observe CTSB activity. The relative fluorescence intensities of CTSB were measured using 44 and 49 COCs. *Asterisk indicates a significant difference between the groups (P < 0.01).

After IVM, the relative fluorescent intensity of CTSB activity in oocytes was higher in the control group than IGF-I treated group (P < 0.05; Figure 3).

## Discussion

The present study, in Experiment I, showed that the number of oocytes recovered by follicular aspiration and ovarian slicing was not significantly different. This is in contrast to the findings by Abdoon (2001), who reported that a significantly higher number of oocytes could be obtained by slicing compared to aspiration in the dromedary camel.

The reason for this discrepancy is not clear, however, as reported earlier, an ovarian structure such as the presence or absence of a corpus luteum seems to influence the number of oocytes available for culture (Abdoon, 2001). Moreover, in the Dromedary, there are no other studies in this regard to shed more light on the matter.

However, in the alpaca (Leisinger et al., 2014), a higher proportion of morphologically normal oocytes were obtained using aspiration compared to the slicing method, though the absolute number of morphologically normal oocytes was higher using the slicing method. Hence, the proportion (and absolute numbers) of oocytes reaching MII after culture was higher when oocytes were retrieved by aspiration. Furthermore, in other domestic species, slicing seems to consistently produce more oocytes than follicular aspiration in cattle (Iwasaki et al., 1987), buffalo (Kumar et al., 1997), and goats (Martino et al., 1994); nevertheless, the developmental competence and viability of oocytes obtained by slicing are lower than those obtained by aspiration.

Moreover, although there was no significant difference between oocytes obtained by aspiration or slicing in terms of completion of maturation, a significantly lower proportion of oocytes were degenerated after follicular aspiration compared to ovarian slicing after 30 h of culture. In cattle, the ability to complete nuclear maturation was higher in oocytes obtained by aspiration compared to slicing (Arlotto et al., 1990). This could be attributed to the fact that slicing will include oocytes from smaller follicles. Oocytes obtained from smaller follicles were found to be less competent to complete nuclear maturation (Leisinger et al., 2014; Martino et al., 1994). On the other hand, in goats, Martino et al. (1994) found no differences in maturation rates between oocytes collected by either of the two methods when stricter oocyte selection criteria were implemented. Khatir et al. (2007) recorded significantly higher cleavage and blastocyst formation rates when oocytes were retrieved from large (>6 mm) follicles compared to oocytes retrieved from smaller follicles (3-6 mm). Based on the results of Experiment 1, aspiration was the chosen method for oocyte collection in subsequent experiments (Experiment II and III).

The camel follicles of different sizes contain IGF-I (El-Bahr et al., 2015). Kafi et al. (2014) measured high concentrations of IGF-I in ovulatory-sized dromedary camel follicles. Moreover, IGF-I elicits anti-apoptotic activity in rabbit embryos (Herrler et al., 1998) and in vitro matured bovine oocytes (Wasielak and Bogacki, 2007). In addition, it has been shown that IGF-1 affects oocyte maturation in several mammalian species, including bovine (Lorenzo et al., 1994; Araujo et al., 2020), rabbit (Lorenzo et al., 1996), human (Gómez et al., 1993), and canine (Sato et al., 2018). It has also been shown that IGF-1 improves in vitro oocyte maturation, fertilization, and embryonic development to the blastocyst stage in mice (Demeestere et al., 2004; Toori et al., 2014). These previous studies coincide with our present study, in which experiment II showed that the IGF-I treatment significantly increased the maturation rate and decreased the degeneration rate after 30 h of IVM compared to that in IVM media without IGF-I. To our best knowledge, this is the first report to demonstrate the positive impact of IGF-1 supplementation during IVM in the camel species. These results suggest that IGF-I improved the quality of oocytes during IVM, as reflected by enhanced cytoplasmic maturation. Moreover, it seems that supplementation with IGF-I mitigates the deleterious effects of IVM-long incubation on the nuclear maturation and subsequent development of bovine embryos.

Previous studies indicated that there was an inverse relationship between CTSB activity and the quality of bovine oocytes and embryos. Therefore, CTSB activity can be used as a marker of inferior quality oocytes and embryos (Balboula et al., 2013; Abdel-Ghani et al., 2019). Our results showed that CTSB activity was significantly lower in MII oocytes treated with IGF-I than those in the control group, indicating that IGF-I supplementation has the ability to improve the quality of MII oocytes.

## Conclusion

The present results demonstrated that a lower degeneration rate in *in vitro* matured oocytes after follicular aspiration might indicate follicular aspiration is the preferred method for oocyte recovery from the camel ovary. Furthermore, the addition of IGF-I during IVM improved the developmental competence of oocytes.

## Data Availability

The data that support the findings of this study are available on request from the corresponding author.
